# Influence of physical activity and mobile phone addiction tendency on depression among Chinese undergraduates

**DOI:** 10.3389/fpsyg.2025.1442707

**Published:** 2025-07-09

**Authors:** Baolin Dong, He Li, Ruqiang Liu

**Affiliations:** ^1^Department of Physical Education, Sanda University, Shanghai, China; ^2^Department of Physical Education, Changchun Finance College, Changchun, Jilin, China; ^3^Department of Physical Education, Suzhou Early Childhood Education College, Suzhou, Jiangsu, China

**Keywords:** undergraduate, physical activity, mobile phone addiction tendency, depression, mediating effect, moderating effect

## Abstract

**Objective:**

This study explored the influence of physical activity (PA) and mobile phone addiction tendency (MPAT) on depression among Chinese undergraduate students, testing the mediating effect of MPAT and the moderating effect of gender.

**Methods:**

The International Physical Activity Questionnaire–Short Form (IPAQ-SF), the Mobile Phone Addiction Tendency Scale (MPATS), and the Center for Epidemiologic Studies Depression Scale (CESD-S) were administered to 2,121 Chinese undergraduates (46.25% male, aged 19.74 ± 1.822 years) from 10 public and private colleges and universities. Analyses on the mediating effects of mobile phone addiction tendency and the moderating effects of gender were conducted using the PROCESS macro (Version 3.5).

**Results:**

There was a significant gender difference in undergraduates' PA (*r* = 0.193, Cohen's *d* = 0.394, *P* < 0.001), while the gender differences in MPAT and depression were not significant (*P* > 0.05). The influence of PA (β = −0.189) and MPAT (β = 0.435) on depression was significant (*P* < 0.001). The mediating effect of MPAT on PA affecting depression was significant, with a mediating effect size of 15.0%. Moreover, gender moderated the influence of PA on depression, and it also moderated the influence of MPAT on depression.

**Conclusion:**

The conclusions corroborate and clarify that MPAT partially mediated the association between PA and depression, and the mediation effects were moderated via gender. This indicates that increasing undergraduates' PA level could effectively avoid MPAT and thus alleviate their depression. Although caution needs to be taken when inferring causal relationships in a cross-sectional design, the present study advances understanding of how undergraduates' PA was related to depression. It also illustrates that educators and parents should pay more attention to undergraduates' PA.

## 1 Introduction

Depression is a common type of persistent negative emotional state, and the World Health Organization noted that it is the second most likely cause of human disability or death (Martin and Cremeens, 2014). In China, undergraduates are a high-risk group for depressive symptoms (Gu, 2014). Clinical and nonclinical experimental studies have shown that although medication or psychological intervention can alleviate the symptoms of depression to some extent, these therapies are not completely effective and have certain limitations and side-effects, or they are associated with recurrence of depression (Hetrick et al., 2017; Maalouf and Brent, 2012). Moreover, adhering to regular and sufficient physical activity can benefit both the human body and mind, and it is an effective treatment method for many psychological disorders (Cleber et al., 2018; Wang, 2021). Self-efficacy theory postulates that mastering a task is one of the strongest sources of self-efficacy (Bandura, 1997). Correspondingly, it may be that individuals could enhance their sense of efficacy and confidence in coping with depression by mastering motor skills and demonstrating motor abilities. Previous research studies have suggested that the degree or frequency of participation in sports is negatively correlated with individual depressive symptoms (Mcdermott et al., 1990; Mcmahon et al., 2017). Moreover, high, medium, and low-intensity physical activity were all found to be associated with an improvement of depressive symptoms, with high-intensity physical activity showing better effectiveness and leading to a higher rate of depressive symptom relief (Hughes et al., 2013). However, several empirical studies have drawn different conclusions, suggesting that physical activity does not have the same functions as antidepressants and cannot directly predict depression but may act indirectly on depression through certain psychological mediators (Chu et al., 2009; Marco et al., 2005). Thus, the following questions arise: can physical activity affect the depression of undergraduates? And what is the intrinsic mechanism in the relationship between physical activity and depression? These issues have not been empirically explored to date.

Recent studies have also found that undergraduates' depression is intricately related to their physical activity and mobile phone addiction tendency. On the one hand, severe mobile phone addiction tendency can lead to negative emotions such as social exclusivity and loneliness, and can even exacerbate depression (Zeng et al., 2022; Ivanova et al., 2020). Behavioral addiction theory postulates that behavioral addiction can lead to many psychological disorders (e.g., depression, anxiety; West and Blackwell, 2015). Although such addiction has not yet been officially recognized by international manuals such as the DSM-5 and the APA, several studies (Demirci et al., 2015; Li et al., 2019) have studied the effect of this phenomenon. Elhai et al. (2016) also observed that people with severe mobile phone addiction tendency tend to center their lives around using phones, but in real life, they might experience depressive symptoms such as low mood and decreased interest in activities (Elhai et al., 2016). On the other hand, several recent empirical studies have found that active and regular physical activity is a feasible strategy for promoting health and preventing diseases, as well as being an effective intervention measure to alleviate the tendency toward mobile phone addiction (Yang et al., 2020). Studies have also found a negative correlation between physical activity and mobile phone addiction tendency among undergraduates: the more physically active they were, the lower the likelihood of mobile phone addiction tendency (Chen and Zhang, 2021; Yang et al., 2019; Kim et al., 2015).

**Figure 1:**
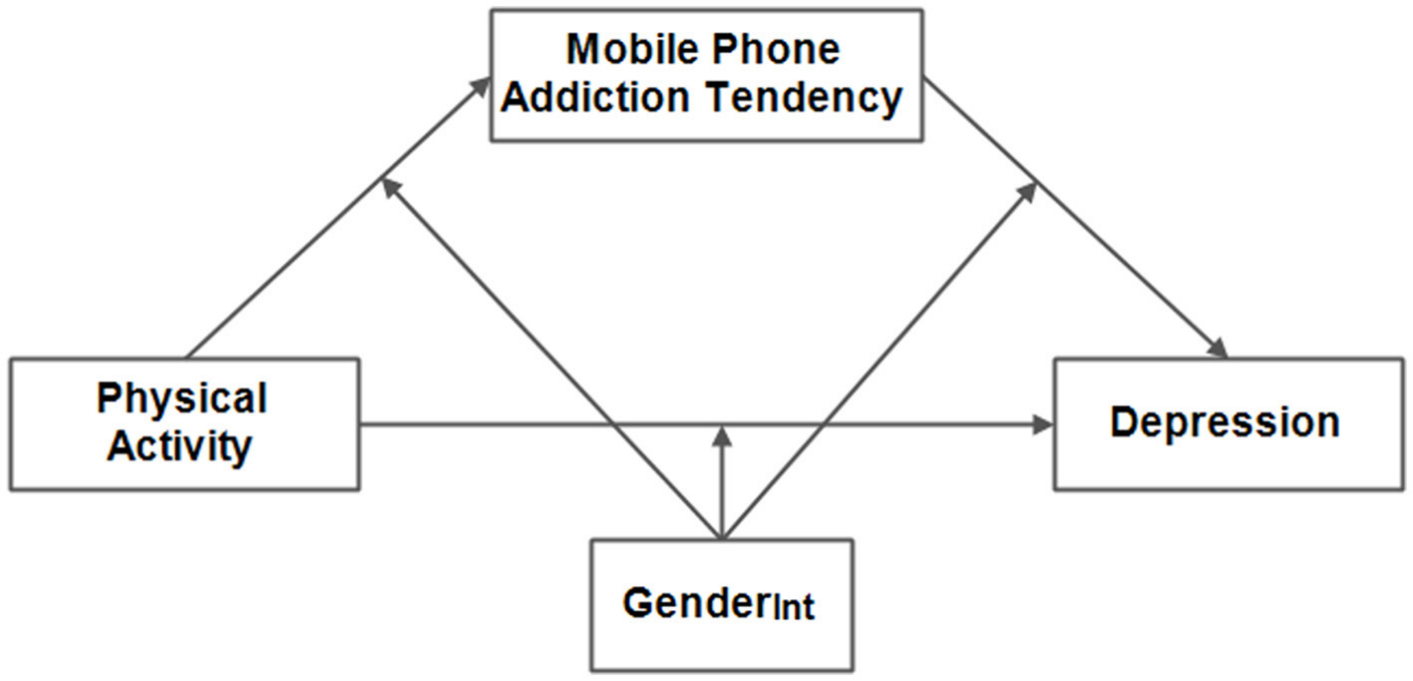
The research hypothetical model. Gender_Int_ = The interaction term between gender and the independent variable.

Previous studies have assessed the independent effect of physical activity or mobile phone addiction tendency on undergraduates' depression (Ivanova et al., 2020; Tao et al., 2020). Nonetheless, fewer studies have comprehensively explored the influence of physical activity and mobile phone addiction tendency on depression among Chinese undergraduates. Stimulus-organism-response (S-O-R) theory postulates that external stimuli could elicit responses through an individual's internal state (West and Blackwell, 2015). Wang (2021) and Xu (2021) observed that one's psychological state plays a mediating role (e.g., sport motivation, subjective exercise experience, mindfulness, and perceived social support) in the relationship between physical activity and depression. More than this, gender socialization theory holds that as children master and internalize the system of “gender,” they learn to recognize attributes, attitudes, and behaviors that are typical of or considered appropriate for each sex (Carter, 2014; Fagot et al., 2012). Chen and Zhang (2021) observed that due to the combined influence of individual social gender, natural gender, and other factors, undergraduates exhibit gender differences in many aspects of behavior, psychology, and emotional experiences (Chen and Zhang, 2021). Therefore, in the present study, a research hypothetical model was constructed and tested (Figure 1). The study aimed to examine the internal mechanism linking physical activity and depression among Chinese undergraduates through empirical research, as well as to explore the mediating effect of mobile phone addiction tendency and the moderated effect of gender. This could be helpful in preventing and addressing many potential problems in the academic and personal lives of undergraduates.

## 2 Materials and methods

### 2.1 Data and participants

Following the sampling principle of combining stratified cluster random sampling with convenience sampling, cities were categorized in this study into two types: provincial capital cities and general cities. Five universities were selected from each type of city, and one class per grade level from each university was selected as the sampling unit. The cross-sectional data presented in this paper were elicited from 2,458 Chinese undergraduates from 10 public and private colleges and universities in Zhejiang Province, Jiangsu Province, and Shanghai in November 2021. Data collection was completed in classroom settings. By scanning a QR code with their phones, participants were able to access the electronic questionnaires. A total of 337 invalid questionnaires were excluded based on criteria for invalid scales, such as missing data on the frequency or time of physical activity of any intensity, testing of the item of reverse scoring, response rates less than 85 percent, and where the answer was clearly inconsistent with the facts. Eventually, 2,121 valid questionnaires were retained. The age range of the survey participants was from 16 to 22 years (three undergraduates did not specify their age), with a mean age of 19.74 years (SD = 1.822 years), and 46.25% of the sample comprised males. The number of students from first to fourth year of university was 519, 534, 598, and 470 respectively. The sample size of this analysis met the social survey sample size standard, determined using a G^*^Power test. The study protocol was approved by the Ethics Committee of Sanda University. Before the survey was administered, written informed consent was obtained from all participants. In addition, 273 undergraduates repeated the survey on November 28, 2021 and December 18, 2021, with a 21-day interval. The final paired sample size was 251.

### 2.2 Measures

#### 2.2.1 Physical activity

Seven items from the International Physical Activity Questionnaire–Short Form (IPAQ-SF) by Meeus et al. were used to measure participants' physical activity (Meeus et al., 2011). Six of the seven items asked participants to specify their level of physical activity during the last seven days, and the remaining item asked them to specify their sedentary time in the working day (such as how many hours and minutes per day). IPAQ-SF was used to investigate the weekly frequency and cumulative time of each physically intense activity. Based on the metabolic equivalent (MET) levels obtained, each physically intense activity was assigned a rate of relative energy expenditure according to the equation provided by Meeus et al. Among them, walking was assigned a MET score of 3.3, medium-intensity physical activity was assigned a MET score of 4.0, and high-intensity physical activity was assigned a MET score of 8.0. Besides these four subscores, a total score was calculated by counting the METs minutes of the first three categories together. Moreover, data cleaning, truncation, abnormal value elimination, and evaluation of physical activity level and grouping were carried out according to the scholars' experience in measurement. The physical activity groupings were as follows: high (i.e., the total duration of various high-intensity physical activities was ≥ 3 days and the total weekly physical activity level was ≥ 1,500 MET min/w, or physical activity of three intensities totaling ≥ 7 days and a total weekly physical activity level of ≥ 3,000 MET min/w); moderate (i.e., various high-intensity physical activities for at least 20 min per day and totaling ≥ 3 days, or various moderate-intensity and/or walking activities for at least 30 min per day and totaling ≥ 5 days, or the total amount of physical activity of three intensities was ≥ 5 days and the total weekly physical activity level was ≥ 600 MET min/w); and low (i.e., no activity reported, or some activities we reported but did not meet the above criteria for medium to high groupings; Fan et al., 2014; Weston et al., 1997). This scale had previously been confirmed by Chinese scholars to have good reliability and validity (Chen and Zhang, 2021). In this study, the Cronbach's coefficient (α) was 0.713, and the stability coefficient of repeated measures was 0.447 (*P* < 0.01).

#### 2.2.2 Mobile phone addiction tendency

Sixteen items from the Mobile Phone Addiction Tendency Scale (MPATS) by Xiong et al. were used to measure participants' internet addiction (Xiong et al., 2012). These sixteen items asked participants to specify their frequency of using smart phones to access the internet (such as browsing websites, chatting, brushing microblogs, playing games) during the past month. This scale has four factors: withdrawal symptoms, salience, social comfort, and mood changes. Responses were coded on a five-point Likert scale ranging from 1 (Not at all) to 5 (Always so), with higher scores indicating a greater severity of internet addiction. This scale had previously been confirmed by Chinese scholars to have good reliability (Cronbach's α was 0.927; Chen and Zhang, 2021). The exploratory factor analysis (EFA) revealed a cumulative contribution rate of 71.156%, KMO=0.948, and the score from Bartlett's sphericity test reached a significant level (Chi Square = 3,085.500, *df* = 120, *P* < 0.001). Confirmatory factor analysis resulted in the following values: *x*^2^/*df*_(98)_ = 3.854, *GFI* = 0.956, *NFI* = 0.980, *IFI* = 0.988, *NNFI* = 0.987, *CFI* = 0.988, *SRMR* = 0.0380, *RMSEA* = 0.067, 90% CI [0.087, 0.108]. In this study, the Cronbach's coefficient (α) was 0.943, and the stability coefficient of repeated measures was 0.597 (*P* < 0.01).

#### 2.2.3 Depression

Twenty items from the Center for Epidemiologic Studies Depression Scale (CES-D) by Radloff (1977) were used to measure depression. These twenty items asked participants to specify how often they had felt or behaved a certain way during the past week, such as feeling they could not “shake off the blues,” even with help from family or friends. Responses were coded on a four-point Likert scale ranging from 1 (Rarely [namely, less than 1 day] or none of the time), 2 (Some or a little of the time, namely 1–2 days), 3 (Occasionally or a moderate amount of time, namely 3–4 days), to 4 (Most or all of the time, namely 5–7 days), with higher scores indicating a greater severity of depression. This scale had previously been confirmed by Chinese scholars to have good reliability (Cronbach's α was 0.874; Chen et al., 2019). Exploratory factor analysis (EFA) revealed a cumulative contribution rate of 64.053%, KMO = 0.956, and the score of the Bartlett's sphericity test reached a significant level (Chi-Square = 4,872.784, *df* = 190, *P* < 0.001). Confirmatory factor analysis resulted in the following values: *x*^2^/*df*_(169)_ = 4.220, GFI = 0.900, NFI = 0.957, IFI = 0.987, NNFI = 0.983, CFI = 0.987, SRMR=0.0472, RMSEA = 0.073, 90%CI[0.096, 0.111]. In this study, the Cronbach's coefficient (α) was 0.961, and the stability coefficient of repeated measures was 0.633 (*P* < 0.01).

### 2.3 Statistical analyses

First, the Mann–Whitney *U* test was used to assess gender differences in the data. Second, each variable (i.e., mobile phone addiction tendency and physical activity) was regressed on depression. Third, analyses on the mediating effects of mobile phone addiction tendency and the moderating effects of gender were conducted using the PROCESS macro (Version 3.5, model 59 for moderated mediation; Hayes, 2013) in SPSS (Version 26.0). Lastly, the formula *R*^2^_*mid*_ = *r*^2^_*MY*_-($R_{\text{Y},\text{MX}}^{2}$-*r*^2^_*XY*_) was used to measure the mediating effect of mobile phone addiction tendency, where *r*^2^_*MY*_ is the square of the correlation coefficient between the mediator variable and the dependent variable, $R_{\text{Y},\text{MX}}^{2}$ is the rate of variation of the overall regression effect of the independent variable and the mediator variable on the dependent variable, and *r*^2^_*XY*_ is the square of the correlation coefficient between the independent and dependent variables (Fairchild et al., 2009).

## 3 Results

### 3.1 Gender differences analysis

The results of the Mann–Whitney *U*-test for gender differences are shown in Table 1. There was a significant gender difference for physical activity (*r* = 0.193, Cohen's *d* = 0.394, *P* < 0.01). In particular, the physical activity level of males (1.29 ± 0.629) was significantly higher than that of females (1.09 ± 0.346).

**Table 1:** Results of Mann–Whitney *U* Test for gender differences.

**Variable**	**PA**	**MPAT**	**Dep**.
Mann-Whitney *U*	7,540.000	7,991.500	8,492.000
*Z*	−3.082	−0.960	−0.204
*P*	0.002	0.337	0.838
Male(M ± SD)	1.29 ± 0.629	41.69 ± 13.303	9.83 ± 10.304
Female(M ± SD)	1.09 ± 0.346	39.82 ± 15.550	12.03 ± 14.621

PA, physical activity; MPAT, mobile phone addiction tendency; Dep, depression.

### 3.2 Correlation analysis

The partial correlation analysis with gender, age and grade as control variables are shown in Table 2. As can be seen, physical activity was related to mobile phone addiction tendency (*r* = −0.544, *P* < 0.001) and depression (*r* = −0.210, *P* < 0.001). Furthermore, there was a significant positive correlation between mobile phone addiction tendency and depression (*r* = 0.442, *P* < 0.001).

**Table 2:** Partial correlation analysis of each variable.

**Variable**	**PA**	**MPAT**	**Dep**
PA	1.000		
MPA	−0.544^***^	1.000	
Dep	−0.210^***^	0.442^***^	1.000

^***^P < 0.001.

PA, physical activity; MPAT, mobile phone addiction tendency; Dep, depression.

### 3.3 Regression analysis

Table 3 shows the results of the regression analyses, with physical activity and mobile phone addiction tendency, respectively, as independent variables and depression as the dependent variable, and with gender, age and grade as control variables. Physical activity had a significant negative impact on depression (*F*_(1,2119)_ = 11.191, β = −0.189, *T* = −3.341, $R_{\text{adj}}^{2}$ = 0.033, *P* = 0.001, 95%CI[0.054, 0.217]). Moreover, mobile phone addiction tendency had a significant positive impact on depression (*F*_(1,2119)_ = 70.081, β = 0.435, *T* = 8.371, $R_{\text{adj}}^{2}$ = 0.186, *P* = 0.000, 95%CI[0.276, 0.446]).

**Table 3:** Regression analysis of the effect of physical activity and mobile phone addiction tendency on undergraduates' depression.

**Variable**	**Dep**								
	**B**	***SE***	β	***T***	***F***	$R_{\text{adj}}^{2}$	***P***	**LLCI**	**ULCI**
PA	−4.946	1.481	−0.189^***^	−3.341	11.161	0.033	0.001	0.054	0.217
MPAT	0.361	0.043	0.435^***^	8.371	70.081	0.186	0.000	0.276	0.446

^***^P < 0.001.

$R_{\text{adj}}^{2}$ = Adjusted *R*^2^.

PA, physical activity; MPAT, mobile phone addiction tendency; Dep, depression.

### 3.4 Moderated mediation analysis

**Figure 2:**
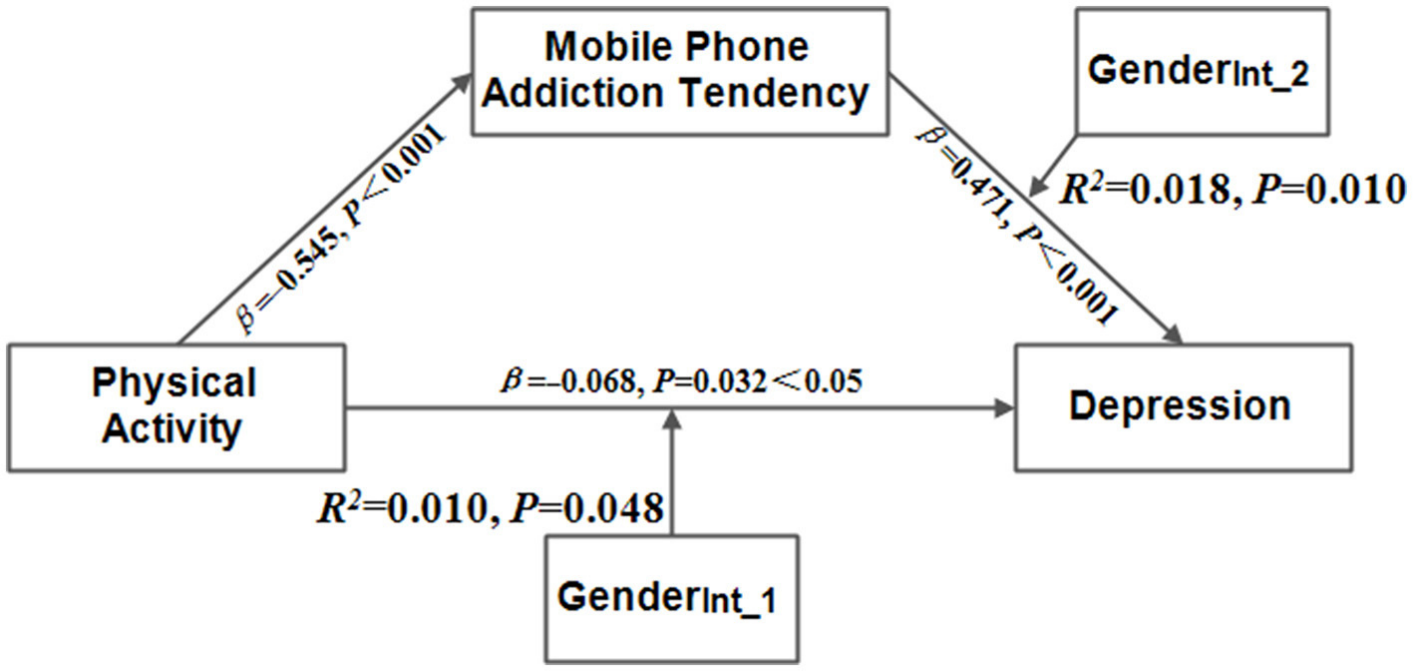
The model of moderated mediation. Gender_Int_1_ = PA × gender, gender_int_2_ = MAPT × gender.

Tables 4, 5, and Figure 2 present the analysis of the indirect impact of physical activity on depression. In Model 1, physical activity had a significant negative impact on mobile phone addiction tendency (*F*_(3,2117)_ = 42.579, 95%CI[−29.358, −9.750], *P* < 0.001), but the moderating effect of gender was not significant (*F*_Int_1(1,2119)_ = 0.200, 95%CI[−4.726, 7.510], *P* = 0.655 > 0.05). In Model 2, physical activity had a significant negative impact on depression (*T* = −1.998, 95%CI[−16.673, −0.305], *P* < 0.05), and the positive impact of mobile phone addiction tendency on depression was significant (*T* = 7.619, *F*_(5,2116)_ = 16.787, 95%CI[0.018, 0.305], *P* < 0.001). In addition, there was a significant positive moderating effect of gender on the impact of physical activity on depression (*F*_(1,2116)_ = 3.940, 95%CI[0.058, 13.486], *P* < 0.05) and on the impact of mobile phone addiction tendency on depression (*F*_(1,2116)_ = 6.730, 95%CI[0.075, 0.544], *P* < 0.01). Furthermore, the mediating effect size of mobile phone addiction tendency was estimated to be 0.150.

**Table 4:** Test of the indirect effects.

**Variable**	**Model 1 (dependent variable: MPAT)**						**Model 2 (dependent variable: Dep)**					
	**Coefficient**	***SE***	***T***	***P***	**LLCI**	**ULCI**	**Coefficient**	***SE***	***T***	***P***	**LLCI**	**ULCI**
Constant	61.619^***^	6.000	10.27	0.000	49.812	73.426	15.981	10.963	1.458	0.146	−5.593	37.555
Gender	−0.089	4.013	−0.022	0.982	−7.985	7.808	−18.068^*^	8.130	−2.222	0.027	−34.069	−2.068
PA	−19.554^***^	4.982	−3.925	0.001	−29.358	−9.750	−1.769^*^	1.619	−1.998	0.049	−16.673	−0.305
MPAT							0.392^***^	0.051	7.619	0.000	0.018	0.305
Int_1	1.392	3.101	0.448	0.655	−4.726	7.510	6.772^*^	3.412	1.985	0.048	0.058	13.486
Int_2							0.309^**^	0.119	2.594	0.010	0.075	0.544
$R_{\text{adj}}^{2}$	0.299						0.320					
*F*	42.579						16.787					

^*^P < 0.05, ^**^P < 0.01, ^***^P < 0.001.

$R_{\text{adj}}^{2}$, Adjusted *R*^2^.

PA, physical activity; MPAT, mobile phone addiction tendency; Dep, depression; Int_1, PA × Gender; Int_2, MAPT × Gender.

**Table 5:** Bootstrap test of the indirect effects.

**Model**	**Interaction term**	***ΔR* ^2^**	***F***	***df_1_***	***df_2_***	***P***	**LLCI**	**ULCI**
1	Int_1	0.001	0.200	1	2,119	0.655	−4.726	7.510
2	Int_1	0.010	3.940	1	2,116	0.048	0.058	13.486
	Int_2	0.018	6.730	1	2,116	0.010	0.075	0.544

Int_1, PA × gender; Int_2, MAPT × gender.

## 4 Discussion

### 4.1 Gender differences

Analysis of the cross-sectional data from 2,458 Chinese undergraduates showed that there was a significant gender difference in undergraduates' physical activity (*r* = 0.193, Cohen's *d* = 0.394, *P* < 0.01), with males demonstrating a significantly higher physical activity level than females. This result is generally consistent with previous research findings (Chen and Zhang, 2021). As is well known, traditional gender role norms assign different codes of conduct and behavior patterns to males and females, and it is generally believed that females should exhibit a gentle and quiet temperament, while males should exhibit traits of independence and competitiveness. Due to this gender role cognition, the behavioral cognition, choices, and habits of males and females during socialization tend to develop in the direction expected by society (Piaget, 1964). Therefore, under normal conditions, in their daily lives, undergraduates exhibit a differentiated pattern whereby males prefer movement and females prefer stillness. In addition, from the perspective of the intensity and content of daily physical activity among undergraduates, the study found that females tended to focus more on low-intensity physical exercise. In contrast, males were relatively energetic and active, often exhibiting a fairly high amount of physical activity in their daily activities and also choosing physical exercise with a certain level of intensity and resistance as their preferred form of physical activity (Yang et al., 2017). In short, gender differences in undergraduates' physical activity may be related to gender-differentiated behavioral patterns and activity content.

The analysis found no significant gender difference between undergraduates' mobile phone addiction tendency and depression. This finding is consistent with previous research findings (Chen and Zhang, 2021; Zhang et al., 2019a,b). There may be two possible reasons for this finding. First, although there may be differences in the preferences and purposes of mobile phone use between males and females (e.g., males preferring to play mobile games and females preferring to shop on apps and watch short videos/live broadcasts), there are similarities in terms of high-frequency loss of control, forced mobile phone use, and emotional responses after withdrawal (e.g., anxiety, unease, and disappointment) among both males and females. This is consistent with the current situation in China, where the proportion of male and female adolescent internet users is basically equal (China Internet Network Information Center, 2021-02-03). Moreover, both males and females inevitably experience difficulties in interpersonal communication or feel pressure in life events. When these challenges are not handled properly or adjusted to in a timely manner, they can lead to a series of similar depressive emotions such as feelings of loss, sadness, and hopelessness. In summary, the gender homogeneity of undergraduates' depression may be related to similar external environmental pressures and life stress stimuli experienced by both males and females.

### 4.2 Effects of physical activity and mobile phone addiction tendency on depression

The findings of the present study indicated that undergraduates' physical activity had a significant negative impact on depression. Previous studies have confirmed that physical activity can reduce the risk of depressive symptoms (Jaworska et al., 2019; Mcdermott et al., 1990; Mcmahon et al., 2017). For instance, Paluska et al. suggested that regular physical activity can quickly divert attention from the sadness and pain caused by stress, thereby reducing symptoms of depression (Paluska and Schwenk, 2000). In other words, engaging in moderate physical activity can enrich undergraduates' positive emotions, enhance their interest in life, and thus alleviate depression. In addition, the study also found that the effect of physical activity on undergraduates' depression was relatively low (*R*^2^ = 0.033). This finding confirms those of previous studies, namely that although physical activity has a certain explanatory power for depression, its antagonistic effect on depression is weak and might operate through other mediators (Hughes et al., 2013; Wang, 2021; Xu, 2021). Furthermore, in terms of physical activity intensity, both males (*M* = 1.29) and females (*M* = 1.09) engaged in a moderate to low level of physical activity intensity. Compared to moderate to high-intensity physical activity, low-intensity physical activity may not have a specific purpose (such as strengthening the body and soothing emotions), and it is mostly a casual low-energy form of body activity (such as simple dynamic behavior or household chores). Therefore, individuals can feel less external pressure, and the intensity of dominant emotional experiences will also be weaker. This viewpoint is supported by the diathesis-stress theory, which posits that there is a dose–response relationship between the stress intensity of life events and the intensity of depression (Hartman et al., 2019; Monroe and Simons, 1991).

The analysis showed that mobile phone addiction tendency had a significant positive impact on depression among undergraduates. Zhou et al. (2010) suggested that depression involves a lack of positive emotions and an increase in negative actions. Mobile phone addiction tendency, as a negative behavior involving non-addictive substances, can lower people's sleep quality, affect their social interaction ability, and lead to feelings of loneliness, alienation, and even reduce self-control and aggravate depression (Tao et al., 2020). In fact, for individuals, mobile phone addiction tendency is often accompanied by an indifference to the surrounding environment, neglect of real life, and confusion and disregard for in-person interpersonal communication. Addicts are prone to lacking the ability to cope with real-life events, often immersing themselves in virtual online worlds or fantasies. This behavior can lead to the development of anxiety and depression (e.g., experiencing anxiety when without access to a mobile phone, compulsive dependence on mobile phones, and panic when trying to quit mobile phone use; Chen et al., 2019; Zhang et al., 2019a,b). In short, mobile phone addiction tendency is a trigger of undergraduates' depression, and the higher level of addiction, the more severe the depression. This viewpoint is supported by behavioral addiction theory, which posits that behavioral addiction can lead to many psychological disorders (e.g., depression; West and Blackwell, 2015).

The analysis also found that mobile phone addiction tendency had a significant mediating effect on the impact of physical activity on depression among undergraduates, with a mediating effect size of 15.0%. This finding confirms those of previous studies, namely that although mobile phone addiction tendency can mediate the effect of physical activity on depression in undergraduates, physical activity might also operate on depression through other mediators (e.g., sport motivation, subjective exercise experience, mindfulness, and perceived social support; Wang, 2021; Xu, 2021). The finding also indicates that intense and regular physical activity of a reasonable intensity could not only help improve undergraduates' social coping ability and social capital but could also help them to avoid mobile phone addiction tendency and improve their physical and mental health. In other words, appropriate physical activity could make people pay more attention to their emotions and experiences, thus reducing the desire for compulsive use of mobile phones; this could act to enhance their sense of joy and happiness in life, mitigating symptoms of depression such as anxiety and feelings of loss. Moreover, intense physical activity could correspondingly reduce the time, frequency, and impulsiveness of mobile phone use in leisure time, thereby enhancing self-identity and self-esteem, increasing confidence in coping with stress events, and alleviating depressive symptoms such as hopelessness and pessimism. Furthermore, adhering to regular and moderate physical activity is an effective way to enhance social communication skills and promote peer relationships, and it could effectively enhance social identity, meet relationship needs, and thus counteract depression. This viewpoint is supported by stimulus-organism-response (S-O-R) theory, which posits that external stimuli (e.g., physical activity) could elicit responses (e.g., depression) through an individual's internal state (e.g., mobile phone addiction tendency; Mehrabian and Russel, 1974).

The Bootstrap method confirmed that gender regulated the impact of physical activity on depression, as well as the impact of mobile phone addiction tendency on depression. Specifically, compared to female students, intense and regular physical activity was more likely to alleviate male students' mobile phone addiction tendency, leading to lower levels of depression. This finding integrates theoretical viewpoints such as coping style theory, gender role theory, and gender socialization theory. Coping style theory postulates that under the stimulation of external stressors, females tend to experience negative emotions through rumination and adopt negative ways to deal with problems, while males tend to consciously shift their attention to neutral or pleasant thoughts and behaviors through distraction and find positive ways to alleviate depression (Gomez-Baya et al., 2017; Nolen-Hoeksema, 1987). Thus, more intense physical activity is more likely to enhance the pleasure and sense of achievement of male students, as well as helping with their self-esteem and relationship needs. They are also more likely to avoid compulsive and impulsive phone dependence behaviors, thereby alleviating depressive tendencies such as feelings of loss and anxiety. In addition, from the perspective of gender role theory and personality traits theory, stereotypes formed by social gender concepts could lead to different behaviors and thinking patterns between males and females (Eagly and Wood, 2012; O'Neil, 1981). Compared to the female students in the sample, the male students were relatively active, and could participate in high-intensity physical exercise. Correspondingly, they were more likely to gain confidence and self-esteem, alleviating depression. In summary, engaging in appropriate forms of physical activity was found to be more beneficial for improving or regulating the lifestyle of male students, helping to reduce negative behavior or feelings of loss of control, and thereby improving emotional experiences and alleviating depression. This viewpoint is supported by gender socialization theory, which posits that from childhood, people learn to recognize attributes, attitudes, and behaviors that are typical of or considered appropriate for each sex (Carter, 2014; Fagot et al., 2012).

### 4.3 Practical implications

The present study advances understanding of how undergraduates' physical activity is related to depression. The conclusions drawn can provide practical reference for formulating physical activity intervention strategies for undergraduates with mobile phone addiction tendency and depression. The findings also illustrate that educators and parents should pay more attention to undergraduates' physical activity.

### 4.4 Limitations

First, this study used a cross-sectional design, which limits the ability to provide strong evidence of causality. Future studies could use longitudinal or experimental research to explore the causal relationship between physical activity, mobile phone addiction tendency, and depression. Second, this study did not analyze whether different intensities of physical activity would lead to differences in the mediating effect. Future studies could explore the differences in the impact of low, medium, and high-intensity physical activity on mobile phone addiction tendency and depression tendencies among undergraduates. Third, this study focused primarily on undergraduates, and more research is needed to explore whether the results apply to other samples, such as children and adolescents. Fourth, the mediating effect of MPAT was reported as 15.0% in this study, which indicates that the effects of physical activity on depression among undergraduates might also be mediated by other psychological factors. Future studies should focus on integrating more variables. Despite these limitations, this study reinforces previous research by revealing the mediating and regulating mechanisms between physical exercise and depression.

## 5 Conclusion

This study found that male Chinese undergraduates were more likely to engage in physical activity than females. Increasing undergraduates' physical activity could alleviate their depression, while mobile phone addiction tendency would serve to increase their depression. Furthermore, improving undergraduates' physical activity level could indirectly alleviate depression through the avoidance of mobile phone addiction tendency. Moreover, compared to females, increasing the amount of physical activity would make it easier to avoid mobile phone addiction tendency among male undergraduates, thus acting to more easily alleviate depression. The present study advances understanding of how undergraduates' physical activity is related to depression. The conclusions drawn can provide practical reference for formulating physical activity intervention strategies for undergraduates with mobile phone addiction tendency and depression. The findings also illustrate that educators and parents should pay more attention to undergraduate students' physical activity.

## Data Availability

The original contributions presented in the study are included in the article/supplementary material, further inquiries can be directed to the corresponding author.
